# Cancer Odor Database (COD): a critical databank for cancer diagnosis research

**DOI:** 10.1093/database/bax055

**Published:** 2017-08-03

**Authors:** Sajjad Janfaza, Maryam Banan Nojavani, Babak Khorsand, Maryam Nikkhah, Javad Zahiri

**Affiliations:** 1 Department of Nanobiotechnology, Faculty of Biological Sciences, Tarbiat Modares University, Jalal Ale Ahmad Highway, Tehran 14117, Iran; 2 Department of Biomaterials, Faculty of Interdisciplinary Sciences, Tarbiat Modares University, Jalal Ale Ahmad Highway, Tehran 14117, Iran; 3 Department of Computer Engineering, Faculty of Engineering, Ferdowsi University of Mashhad, Mashhad, Iran; 4 Bioinformatics and Computational Omics Lab (BioCOOL), Department of Biophysics, Faculty of Biological Sciences, Tarbiat Modares University, Jalal Ale Ahmad Highway, Tehran 14117, Iran

## Abstract

Here, we present Cancer Odor Database (COD), a web-based database comprising comprehensive information of volatile organic metabolites of cancer (VOMC), known as cancer odor, which gives a structured overview of VOMCs that are of critical importance in cancer research. The database contains more than 1300 records with 19 critical features for each record, such as structural and chemical properties (e.g. boiling point, molecular formula and molecular weight) of 450 different VOMCs and their origins, which can be used effectively to identify correlations between VOMCs and various types of cancer. COD database has been constructed based on the data that were directly extracted from literature. COD information can be helpful for cancer researches, especially for those who are developing sensors and electronic nose systems for cancer detection. COD is freely available for non-commercial purposes online at http://bioinf.modares.ac.ir/software/cod.

**Database URL:** http://bioinf.modares.ac.ir/software/cod/

## Introduction

Cancer is considered as a complex genetic disease characterized by the uncontrolled growth and the spread of abnormal cells. Diagnosis and therapy of various types of cancers have received a lot of attention over the past few decades (1,2).

Despite these efforts, cancer remains a serious health problem and a major cause of death worldwide. Therefore, there is a need for development of new approaches for cancer detection and treatment, like finding novel biomarkers. Discovery of cancer biomarkers that are specific to or associated with cancer such as tumor-derived microRNAs (3), DNA methylation (4) and tumor-associated antigens (5) holds promising future for novel diagnostic and therapeutic targets of cancer (6).

According to the fact that cancer is also considered as a metabolic disease (7), recently many attempts have been made to determine relevant metabolic targets. It has been well known that the metabolic profile of cells changes during cancer and many metabolic pathways can be affected by or, affect the cancer development (7). For instance, malignant cells prefer to metabolize glucose via aerobic glycolysis rather than oxidative phosphorylation (known as Warburg effect) (8,9) which results in change in the cell metabolic profile. Therefore, this altered metabolic profile can be considered as a hallmark of cancer.

Volatile organic compounds are among the most common metabolic products generated during metabolic or pathological processes. These volatile molecules with a low boiling point and high vapor pressure give different odor to the biological samples (10). There are a number of studies which have investigated volatile organic compounds (e.g. methanol) in healthy human specimens (e.g. breath) (11).

A considerable body of research has demonstrated the concentration profile of volatile organic metabolites released from body fluids, such as blood, urine, and saliva changes during various diseases (12). These gas phase biomarkers, reflecting disease states and physiological processes in the body, offer the potential application in early detection of numerous diseases (12). For instance, several studies have investigated the ability of diagnosis and follow-up of Alzheimer's (13,14), Parkinson's (14), infectious (15) and respiratory (16) diseases via the analysis of volatile organic metabolites.

**Figure 1.**
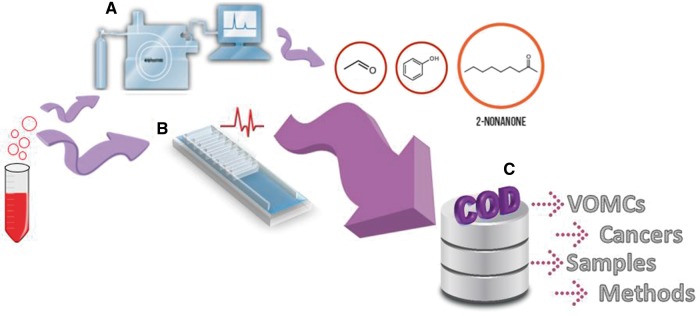
Two ways of cancer detections based on odor of biological samples: (A) analytical instruments such as gas chromatography mass spectrometry (GC/MS) which is the most commonly method used for separation and identification of VOMCs, (B) Sensors and electronic nose measurements. COD, a useful web-based database, collects whole information reported on VOMCs (C).

Specially, the relationship between volatile organic metabolites and cancer has been intensively investigated recently, and a number of compounds have been reported as potential biomarkers for different cancers (10). Later, many investigations focused on the detection of various types of cancers based on VOMCs with the use of animals (e.g. dogs and mice) (17), electronic nose and other sensitive analytical instrumentation (10) (Figure 1A and B).

However, despite many reports on VOMCs and significant progress in this field, it still faces some challenging problems. For instance, analysis of VOMCs might be affected by some external or internal factors including age, sex, genetic, geographical variation and lifestyle (18,19).

Another major limitation in this field is that each work only refers to its results or other limited works and there are no comprehensive resources that provide the detailed information about VOMCs.

Here we present the COD database, which provides detailed and valid information about VOMCs. COD database enables the researchers to do comparison among the changes of VOMCs in different cancers (Figure 1C).

## Database description

### Data collection

Comprehensive information of cancer-related volatile organic metabolites that are produced, catabolized or their concentrations are changed in various types of cancers is collected in the COD database. The aim of the project is to facilitate search, analysis and investigation of VOMCs and provide general overview of research in this context.

Candidate papers were found via searches in various search engines including Web of Knowledge, Google Scholar and PubMed, covering more than thousand articles. Among >1000 papers, 104 were selected by applying the following criteria: (i) peer-reviewed studies published in English language journals (ii) having a primary focus on VOMCs. The required information was directly extracted from the selected publications and manually curated with experts to minimize errors.

To complete the VOMCs data, information about chemical and physical properties of VOMCs was obtained from PubChem database (http://pubchem.ncbi.nlm.nih.gov) and integrated to the COD. All the related publications for any search results will be presented with the detailed information.

**Figure 2.**
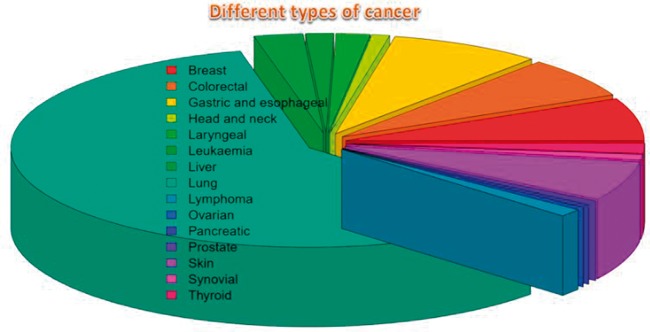
The pie chart of different types of cancers in the database.

COD contains comprehensive information of VOMCs reported in different types of cancers including lung, colorectal, gastric, esophageal, ovarian, bladder, breast, prostate, head and neck, pancreatic, skin, synovial, laryngeal, liver, leukemia, and lymphoma. The pie chart shows the proportions of the results for each cancer type (see Figure 2).

### Database implementation details

The interface of COD has been implemented by html5 and CSS. Also, using JavaScript, a simple keyword-based search interface is added to access the data and make ease in entering the input search keywords.

The server side scripting has been done using PHP on a MySQL backend which allows everyone to easily access each item of the database and download it in a tab separated format text.

### Web interface

**Figure 3.**
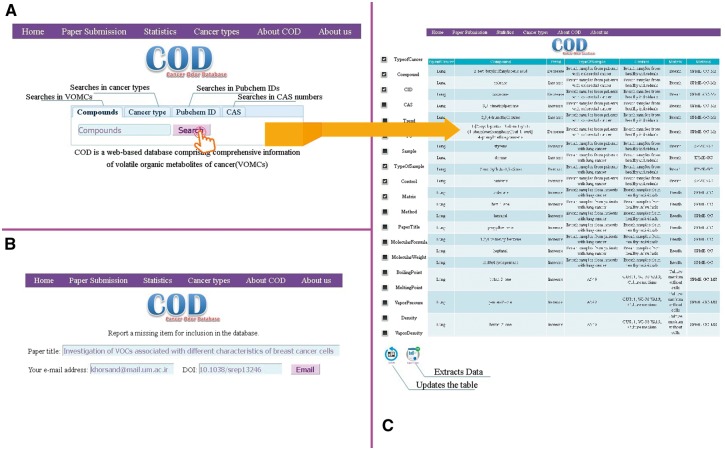
The web interface of the COD database: Home page (A), paper submission page (B) and results (C).

COD is a web-based resource focused on VOMCs and freely accessible at http://bioinf.modares.ac.ir/software/COD. To provide easy access to data, the database offers a variety of options for searching and displaying the results, which implemented with a user-friendly interface. Figure 3 demonstrates an illustrative screenshot of the COD interface.

### Search section

The database provides flexible search options. Users can search in certain datasets by defining the data type using four tabs. Selecting the ‘Compounds’, ‘Cancer type’, ‘Pubchem ID’ and ‘CAS’ sections enables searching among the VOMCs, cancer types, Pubchem ID and CAS numbers, respectively.

### Results section

There is one row for each record in the results page and the records are represented in several columns in which following descriptors are presented: cancer types, VOMCs trend and its fold of changes in certain cancer, properties of biological samples including type of sample, experimental conditions (*in vivo* or *in vitro*), extraction conditions, type of sample used as control, method of analysis and chemical information of compounds including IUPAC name, other names, CID (A compound identifier for a unique chemical structure in the PubChem database), chemical structure, boiling point and molecular weight of VOMCs. Also, details of the related publication are given for each record. Users can add or remove one or more parts of the mentioned descriptors to or from demonstrated search results.

The presence of CID and CAS number for each compound let the user to get more chemical information about VOMCs from other databases like PubChem, if needed.

In addition, entire or certain parts of database with desirable sorting can be downloaded via the ‘Export data’ button. By the data submission page on the website, researchers are able to inform us of the new publications.

**Figure 4.**
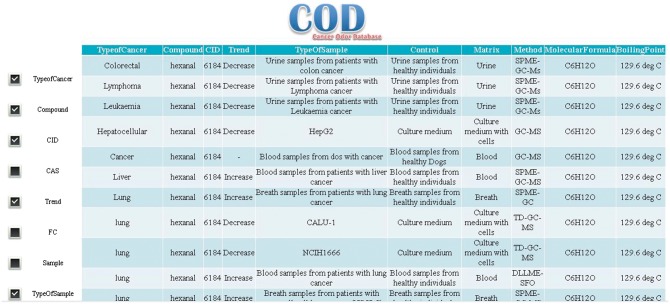
Results of searching the database for hexanal.

To examine the effectiveness of our database, we used ‘hexanal’, a potential biomarker for several cancers (20,21), as a case study. ‘hexanal’ was searched in ‘Compounds’ section. Our system shows all types of cancers (25 results) in which hexanal is produced, metabolized, or its concentration is changed (Figure 4). Based on the results, hexanal has been reported for 20 *in vivo* (including urine, blood and breath) (22), one *ex vivo* and four *in vitro* samples in five different types of cancers including colorectal (22), lymphoma (22), leukemia (22), liver (23), lung (24), gastric (25) and breast (26) cancers.

Users also can search in cancer types. For instance, when looking for VOMCs in a specific type of cancer such as lung cancer, COD returns 751 search results about VOMC with detailed information e.g. IUPAC name, CAS number, molecular formula, trend of compound in cancer, fold of change (FC), type of sample, type of control and other useful information related to the cancer of interest.

VOMCs trend of change can be found in the ‘Trend’ field. During the cancer, a VOMC can be produced, catabolized, or its concentration is changed (increase or decrease). FC or fold of change shows how much a molecule is changed in cancer sample compared to control. FC is very important parameter for selecting a biomarker. VOMCs with higher FC are preferred to be used as biomarkers.

Columns ‘matrix’ and ‘type of control’ refer to biological matrix and control used in the experiment for VOMCs identification, respectively.

Matrix indicates the type of sample (e.g. urine, blood, cell culture medium and breath) used for VOMCs detection, in the corresponding experiment. Knowing the matrix properties is very important for researchers who want to study, interpret or analyze the results.

Another parameter that might be important for researchers is the techniques that have been used in the characterization of VOMCs. This parameter is indicated as ‘method’ in the results table.

This above-mentioned information is particularly useful for scientists investigating VOMCs, and also for researchers that work on development of sensors and electronic nose systems for cancer detection.

## Conclusion and perspectives

Volatile organic metabolites are produced in metabolic processes in both cancer and normal cells. It has been experimentally confirmed that the pattern of VOMCs’ changes during cancer and the analysis of them can differentiate between cancer patients and healthy controls.

So far, VOMCs of different types of cancers including lung, breast, prostate, colorectal, gastric, esophageal, ovarian, bladder, pancreatic, skin, synovial, laryngeal, liver, leukaemia, lymphoma, and, head and neck have been investigated as a frontier for cancer diagnosis and the reliability of this approach in detecting cancer has been demonstrated.

On the other hand, there are many reports regarding VOMCs in the literature which most of them are limited to the certain types of cancer. So, it is not possible to do a comprehensive analysis on the results of a large number of publications without having a structured database. COD database is developed by bringing all the information in this field together to address this problem and give an extensive overview of cancer-related volatile molecules.

The manually curated data, which were extracted from the literature, ensures that the collected data are accurate and reliable. Although COD seems to be a small database, strength of the database lies in its quality and critical applications in cancer research.

Providing chemical and physical information of VOMCs obtained from PubChem makes it possible to investigate and compare chemical and physical properties of VOMCs in different cancers. All reported studies in the literature have their own pre-concentration, separation and identification methods with different experimental condition. In addition, various types of patient samples such as urine and breath of a certain cancer often possess different VOMCs profile.

According to the published studies, it seems that each analytical method alone is able to identify only some of the available VOMCs in the samples. In addition, by using different samples from the same cancer different VOMCs have been reported for the same cancer. Therefore, a source that can collect the entire information of VOMCs in various experimental conditions will be particularly helpful.

The COD database is developed to help researchers investigate VOMCs easily. Data can be used for developing new sensing tools (e.g. electronic nose) and analyzing information as well as mining data for testable hypotheses. We hope COD can help to improve the ability to diagnose, treat and prevent cancer.

## Availability

The COD database will be continuously maintained and updated. The database is now publicly accessible at http://bioinf.modares.ac.ir/software/COD.
